# Early post-operative hydrocephalus following retrosigmoid vestibular schwannoma resection: Incidence, clinical patterns, and outcomes

**DOI:** 10.1016/j.bas.2025.105598

**Published:** 2025-09-09

**Authors:** Kamil Krystkiewicz, Marcin Tosik, Marcin Birski, Łukasz Szylberg, Jacek Furtak, Marek Harat

**Affiliations:** a Department of Neurosurgery and Neurooncology, Copernicus Memorial Hospital, Łódź, Poland; b Department of Neurosurgery, 10th Military Research Hospital, Bydgoszcz, Poland; c Department of Tumor Pathology and Pathomorphology, Oncology Center-Prof. Franciszek Łukaszczyk Memorial Hospital, Bydgoszcz, Poland; d Department of Obstetrics, Gynaecology and Oncology, Collegium Medicum in Bydgoszcz, Nicolaus Copernicus University in Torun, Bydgoszcz, Poland; e Medical Faculty of the University of Science and Technology in, Bydgoszcz, Poland

**Keywords:** Hydrocephalus, Obstructive hydrocephalus, Vestibular schwannoma, Retrosigmoid approach, External ventricular drainage

## Abstract

**Introduction:**

Early post-operative hydrocephalus after retrosigmoid vestibular schwannoma (VS) resection is recognised but insufficiently characterised.

**Research question:**

What are the incidence, clinical phenotypes, peri-operative predictors, and outcomes of early post-operative hydrocephalus?

**Material and methods:**

We retrospectively reviewed 116 consecutive adults who underwent primary retrosigmoid VS removal between 2020 and 2024. Pre-, intra-, and early post-operative variables were correlated with symptomatic hydrocephalus, defined as ventriculomegaly plus neurological decline within four post-operative days. Management, length of stay (LOS), and late CSF-diversion requirements were analysed.

**Results:**

Symptomatic hydrocephalus occurred in eight patients (6.9 %) and required external ventricular drainage (EVD) in six (5.2 %). Two reproducible phenotypes were observed: an acute course (n = 2) with abrupt Glasgow Coma Scale (GCS) < 12, driven by tumour-bed hematoma or fulminant cerebellar edema; EVD was maintained for 11–14 days, one patient died, the other needed a ventriculoperitoneal shunt; a mild course (n = 6) with GCS 13–14 and ipsilateral cerebellar edema; four patients required 5-day EVD, all recovered fully. Tumour-bed hematoma was the only significant predictor (*p* = 0.0018); demographics, tumour size/volume, cystic component, and extent of resection were neutral. EVD placement prolonged median LOS (20 vs 13 days, *p* = 0.001).

**Discussion and conclusion:**

Early post-operative hydrocephalus complicates 5 % of retrosigmoid VS resections and manifests as either a life-threatening acute form or a mild form. Vigilant early MRI/CT and a stepwise escalation from steroids to selective EVD achieve favourable outcomes; preventing tumour-bed hematoma is the principal modifiable risk factor.

## Highlights

• Hydrocephalus in 5 % of retrosigmoid VS cases, always within 4 days post-op.
•Two forms: mild (GCS ≥13) heals with steroids/EVD; acute (GCS <12) riskier.
•Tumour-bed hematoma was the sole predictor; size, cysts, age were neutral.
•Early MRI/CT, drugs, and selective EVD kept shunt rates low and drains safe.
•42 % had silent ventriculomegaly, so action must follow symptoms, not ventricle size.

## 1 Introduction

Hydrocephalus is a common clinical sign of vestibular schwannoma (VS) and can be either obstructive or communicating (Rogg et al., 2005; di Russo et al., 2021; Pirouzmand et al., 2001). In the past, obstructive hydrocephalus was more common and easily explained by compression of the cerebral aqueduct or fourth ventricle outlets. Widespread magnetic resonance imaging now shows ventricular enlargement without clear obstruction in 1.2–42 % of cases (Al et al., 2013; Fukuda et al., 2007; Gerganov et al., 2011; Samii and Matthies, 1997). The management of such preoperative hydrocephalus is debated: some experts prefer cerebrospinal fluid (CSF) diversion before tumor removal, while others go directly to resection (di Russo et al., 2021). Available diversion methods include ventriculoperitoneal shunt (VPS), external ventricular drainage (EVD), and endoscopic third ventriculostomy.

A distinct entity—postoperative hydrocephalus—may occur following VS resection, often triggered by cerebellar edema or a tumor-bed hematoma that temporarily blocks CSF outflow (Briggs et al., 1993). Samii et al. reported an incidence of 2.3 % (Samii and Matthies, 1997), yet detailed data on early clinical progression, risk factors, and long-term outcomes are limited. This study examines explicitly early postoperative hydrocephalus after retrosigmoid VS resection, analyzing perioperative risk factors, clinical-radiological patterns, management, and outcomes, and proposing a practical treatment algorithm.

## 2 Methods

### 2.1 Study design and data source

A retrospective review was conducted of all adults (≥18 years old) who underwent primary retrosigmoid craniotomy or craniectomy for VS at a single tertiary center between January 2020 and June 2024. Exclusion criteria included translabyrinthine or middle fossa approaches, staged or revision surgeries, and incomplete imaging or <12-month follow-up. Cases were identified using ICD-10 code D33.3.

Recorded variables included age; sex; tumor size and volume (Brainlab Cranial v4.0); cystic component; pre- and post-operative cerebellar/brainstem edema; aqueduct or fourth ventricle obstruction; ventricular size (Evans index); extent of resection (Hannover grading); clinical signs of raised intracranial pressure; length of stay (LOS) across all wards; CSF diversion procedures; and related complications.

All patients underwent contrast-enhanced at least 1.5-T MRI (T1 pre- and post-gadolinium, T2, FLAIR) within 24–72 h postoperatively, or a non-contrast CT scan when MRI was contraindicated. Imaging was independently reviewed by two authors (K.K., M.T.). Ventricular enlargement was defined as the Evans index >0.30. CSF diversion by EVD was documented, including timing, duration, and complications. Long-term follow-up was conducted in an outpatient clinic. The institutional ethics committee waived informed consent due to the study's retrospective nature.

### 2.2 Statistical analysis

Continuous variables were first assessed for normality with the Shapiro–Wilk test. Data that met the assumption of normality are reported as mean ± standard deviation and compared between groups using the independent-samples Student's *t*-test; non-normally distributed data are summarised as median (inter-quartile range, IQR) and analysed with the Mann–Whitney *U* test. Categorical variables are shown as counts (percentages). Associations between categorical predictors and early post-operative hydrocephalus were examined with Pearson's χ^2^ test; when the expected frequency in any cell was <5, Fisher's exact test was applied. Effect sizes are expressed as mean differences with 95 % confidence intervals (CI) for continuous variables and odds ratios with 95 % CI for categorical comparisons. All statistical tests were two-sided, and *p* ≤ 0.05 was considered significant. Analyses were executed in IBM SPSS Statistics, version 28 (IBM Corp., Armonk, NY, USA).

## 3 Results

### 3.1 Patient and tumour characteristics

Between January 2014 and June 2020, 116 patients met the inclusion criteria. The cohort included 74 women (64 %) and 42 men (36 %), with a mean age of 47.3 ± 13.9 years. Mean tumor dimensions were 31.4 ± 11.4 mm anteroposteriorly, 28.8 ± 9.8 mm mediolaterally, and 28.6 ± 9.9 mm craniocaudally, with a mean volume of 13.7 ± 10.3 cm^3^. Tumors were on the right side in 63 patients (54 %) and on the left side in 53 (46 %). According to the Hannover classification, three tumors (2.5 %) were grade T1, three (2.5 %) T2, seven (6 %) T3a, seven (6 %) T3b, 21 (18 %) T4a, and 67 (65 %) T4b. The mean operative time was 4 h 44 min ±1 h 37 min; the mean length of stay (LOS) was 13.9 ± 9.8 days (range 6–76). The mean follow-up period was 21.8 ± 11.8 months.

Peritumoral T2/FLAIR hyperintensity suggestive of edema was observed in 27 patients (23 %). Four patients (3 %) had pre-existing VPS, and one (0.8 %) had an endoscopic third ventriculostomy.

### 3.2 Hydrocephalus

#### 3.2.1 Pre-operative status

Hydrocephalus was present preoperatively in 18 patients (16 %), with 11 cases being obstructive (9 %) and seven communicating (6 %). The mean Evans index for the entire cohort was 0.28 ± 0.05, increasing to 0.33 ± 0.03 in hydrocephalic cases.

#### 3.2.2 Post-operative changes

Post-operatively, ventricular enlargement occurred in 14 patients (12 %). Eight (7 % of the cohort) developed impaired consciousness, whereas six were asymptomatic and ventriculomegaly was detected only on routine imaging. Headache, nausea and vomiting were non-specific and occurred in both groups.

#### 3.2.3 Two clinical patterns emerged

• Group 1 – mild course (n = 6). Modest consciousness decline (GCS 13–14).
• Group 2 – acute course (n = 2). Abrupt deterioration to GCS <12.

**Fig. 1:**
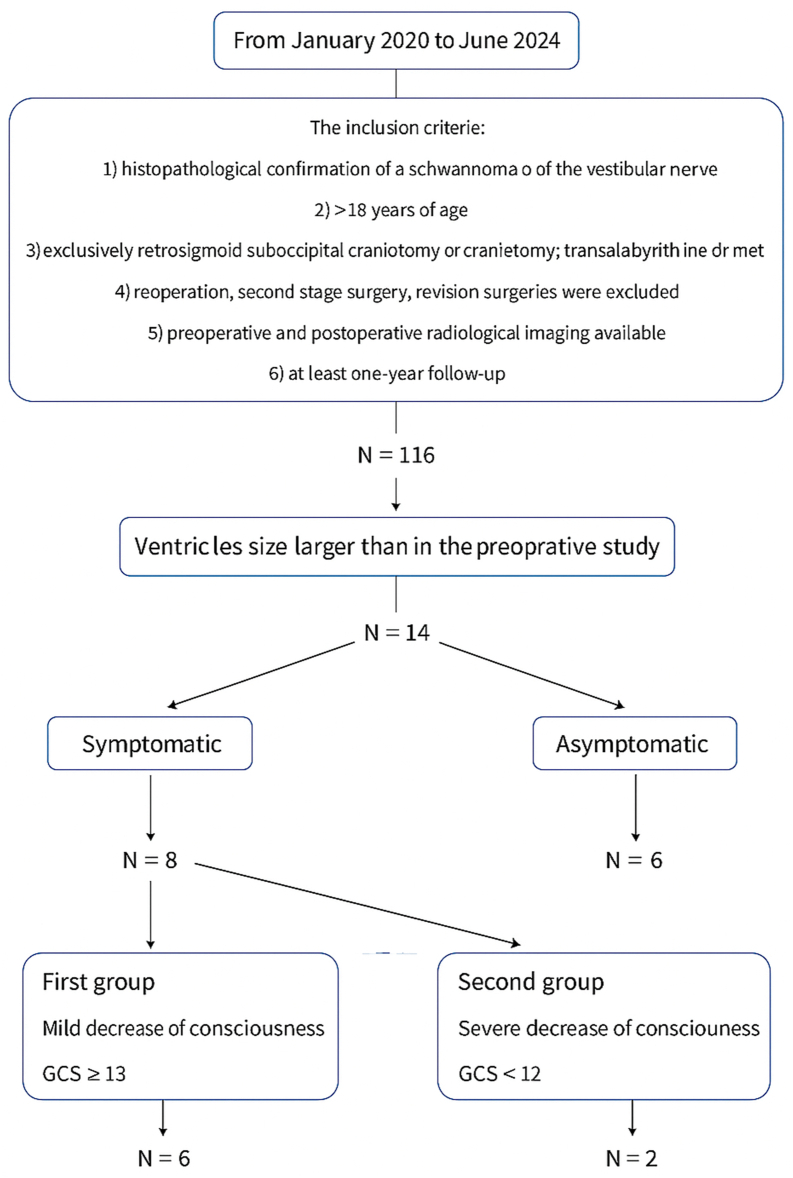
Study flow-chart depicting case selection and clinical stratification. GCS – Glasgow Coma Scale.

A flowchart of the case selection is presented in Fig. 1.

#### 3.2.4 Group 1 – mild course

**Fig. 2:**
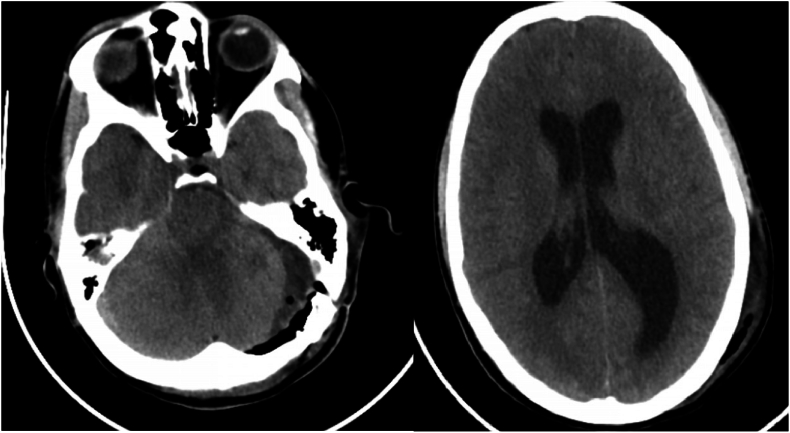
Example of the first group of postoperative hydrocephalus. Note mild edema in the left cerebellar hemisphere, compressed 4th ventricle, and enlarged lateral ventricles.

All six patients exhibited ventriculomegaly, but the convexity CSF spaces remained open, and no periventricular lucency was observed. MRI on POD 1 revealed ipsilateral cerebellar hemisphere edema with fourth ventricle effacement (Fig. 2); there was no hematoma. Two patients (33 %) responded to dexamethasone and mannitol alone; four required EVD insertion—two on postoperative day 2, one on day 3, and one on day 4. All improvements were immediate; the drains were removed on POD 5 after 24 h of clamping. No procedure-related complications occurred, and none needed further CSF diversion.

#### 3.2.5 Group 2 – acute course

**Fig. 3:**
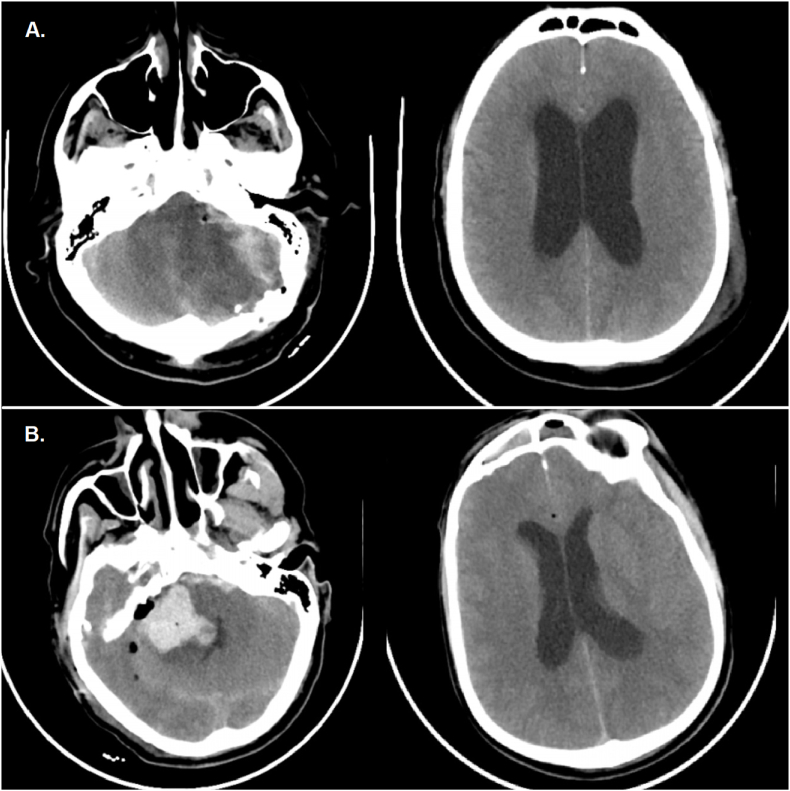
A. Case of the acute postoperative hydrocephalus classified as the second group. Note the marked edema and hemorrhagic changes in the left hemisphere associated with the venous stroke. B. Hematoma in the post-resection cavity. The 4th ventricle is compressed but still visible.

Both patients showed collapsed convexity CSF spaces, periventricular lucency, and a large posterior fossa mass effect. Case 1: Several hours after extubation, the patient became comatose. CT revealed a large tumor-bed hematoma causing obstructive hydrocephalus (Fig. 3). Emergency hematoma evacuation and EVD insertion restored neurological function; however, persistent communicating hydrocephalus required VPS placement after 14 days. Case 2: On POD 1, the patient developed coma and respiratory arrest. CT showed cerebellar edema, intraparenchymal hemorrhage, and obstructive hydrocephalus. Despite emergency posterior fossa decompression and EVD placement, the patient died after 14 days in intensive care.

Table 1 lists the characteristics of the eight patients who required EVD.

**Table 1:** Detailed analysis of patients who needed external ventricular drainage implantation. ∗ - postoperative day.

	Sex	Age	Side	Hannover	Tumor volume (cm^3^)	Evans preop.	Obstruction	Edema preop.	Cysts	Degree of resection	Evans postop.	Edema postop.	Hematoma	Decreased consciousness	POD∗	How long drainage (days)	Opening pressure (cm H20)	Shunt?
1.	F	57	Left	4b	12.7	27 %	No	No	Yes	99 %	29 %	1	No	2	3	5	21	No
2.	M	28	Left	4b	17.76	27 %	No	Yes	Yes	98 %	30 %	1	No	2	2	5	19	No
3.	M	54	Left	4b	2.39	37 %	Yes	Yes	No	76 %	40 %	1	Yes	1	1	14	35	No
4.	M	34	Right	4b	35	25 %	No	No	Yes	97 %	26 %	1	Yes	1	1	14	40	Yes
5.	F	55	Left	4a	5.73	22 %	No	Yes	No	99 %	25 %	1	No	1	2	5	25	No
6.	F	56	Right	4a	16.3	28 %	No	No	Yes	98 %	30 %	1	No	1	4	5	20	No

#### 3.2.6 Risk factors

Univariate analysis revealed no association between early postoperative hydrocephalus and age, tumor size or volume, sex, preoperative Evans index, extent of resection, peritumoral edema, aqueduct obstruction, cystic morphology, or prior radiosurgery (Table 2). Of five patients previously treated with Gamma Knife, three (60 %) developed ventriculomegaly, but none were symptomatic.

**Table 2:** Comparison between groups that did not have external ventricular drainage (EVD) placed and those that required EVD placement. GTR – gross total resection. NGTR – near gross total resection. ST – subtotal resection.

Variable	No EVD	EVD	Statistical analysis
	Average	Average	p
Length of hospitalization (days)	13.4 ± 9.8	19.6 ± 6.4	**0.0019** (U Mann-Whitney test)
Tumor volume (cm^3^)	11.1 ± 10.2	15.2 ± 9.9	0.24 (U Mann-Whitney test)
Age (years)	47.5 ± 10.0	44.5 ± 12.0	0.59 (U Mann-Whitney test)
Anterior-posterior distance of tumor (mm)	30.78 ± 11.8	35.12 ± 5.91	0.27 (U Mann-Whitney test)
Left-right distance of tumor (mm)	28.6 ± 10.1	28.12 ± 6.96	0.88 (U Mann-Whitney test)
Superior-inferior distance of tumor (mm)	28.1 ± 10.2	32.12 ± 6.96	0.29 (U Mann-Whitney test)
Preoperative Evans ratio	0.28 ± 0.04	0.27 ± 0.05	0.70 (U Mann-Whitney test)
Degree of resection (%)	86 ± 22	86 ± 21	0.41 (U Mann-Whitney test)
Sex	Male – 39 (35 %) Female – 71 (65 %)	Male – 3 (50 %) Female – 3 (50 %)	p = 0.21, ꭓ^2^
Hannover scale	T1 – 3 (2 %) T2 – 3 (2 %) T3a – 7 (3 %) T3b – 7 (4 %) T4a – 19 (14 %) T4b – 64 (75 %)	T4a – 2 (33 %) T4b – 4 (67 %)	p = 0.95, ꭓ^2^
Cystic tumor	Yes – 23 (21 %) No – 87 (79 %)	Yes – 4 (67 %) No – 2 (33 %)	p = 0.18, ꭓ^2^
Obturation	Yes – 26 (24 %) No – 84 (76 %)	Yes – 1 (17 %) No – 5 (83 %)	p = 0.33, ꭓ^2^
Cerebellar edema	Yes – 26 (24 %) No – 84 (76 %)	Yes – 3 (50 %) No – 3 (30 %)	p = 0.21, ꭓ^2^
Extent of resection	GTR – 21 NGTR – 8 ST - 81	GTR – 0 NTGR – 0 ST - 6	p = 0.13, ꭓ^2^
Preoperative radiosurgery	5	0	p = 0.76, ꭓ^2^

Only two variables correlated with EVD placement: postoperative ventricular size (χ^2^, *p* = 0.0015) and tumor-bed hematoma (χ^2^, *p* = 0.0018). Patients requiring EVD had a longer LOS than conservatively managed patients (median 20 vs 13 days; Mann–Whitney *p* = 0.0019).

## 4 Discussion

Although pre-operative hydrocephalus in vestibular schwannoma (VS) has been examined extensively, robust data on the early post-operative phase remain scant. Huang et al. reported acute hydrocephalus in 1.2 % of 1176 patients, but offered no details on clinical course or outcome (Huang et al., 2017). Using an administrative database, Mahboubi et al. found a 3.6 % incidence, yet their analysis lacked granular radiological and neurological information (Mahboubi et al., 2014). Our rate of 6.9 % is therefore at the upper end of published figures, likely reflecting a systematic policy of early MRI/CT and prospective neurological scoring that captured even transient, mild cases.

Post-operative ventricular enlargement alone proved unreliable: 42 % of patients with enlarged ventricles were neurologically intact and responded to steroids plus mannitol without CSF diversion. This highlights that ventricle size, although easy to quantify, should be interpreted in conjunction with clinical signs of raised intracranial pressure (ICP) and posterior fossa imaging. Our data support the classic neurosurgical principle that the clinical phenotype takes precedence over imaging when deciding on intervention.

All symptomatic cases were obstructive, underlining the dominant role of posterior-fossa mass effect. Two mechanistic patterns emerged. The first - mild course – cerebellar-hemisphere edema effacing the fourth ventricle, but preserving supratentorial compliance, resulting in slow GCS drift (13–14) and reasonable response to short-term EVD. The second – acute - rapid aqueductal occlusion from tumour-bed hematoma or fulminant edema, producing collapsed convexity cisterns, periventricular lucency and potential herniation. Outcome hinged on prompt decompression; one patient survived with delayed VPS, whereas the other died despite maximal therapy. The divergence mirrors experimental work showing that the rate of ICP rise—rather than absolute ventricle size—dictates cerebral perfusion compromise.

Tumour bed hematoma was the sole perioperative variable associated with hydrocephalus (χ^2^ *p* = 0.0018). Intraoperative haemostasis, judicious blood pressure control, and early imaging, therefore, remain critical. Peritumoural T2/FLAIR edema (PTE), identified in 23 % of patients, did not correlate with hydrocephalus, contradicting the hypothesis that VEGF-driven edema predisposes to CSF pathway obstruction. Similarly, neither patient demographics nor tumor dimensions or extent of resection showed any association with early postoperative hydrocephalus, indicating that post-closure events within the posterior fossa, rather than preoperative characteristics, are the primary determinants of risk. We concur with Samii et al. (2015) that meticulous preservation of the arachnoid plane and bridging veins mitigates venous infarction and late swelling; however, this requires validation in a multi-centre setting.

**Fig. 4:**
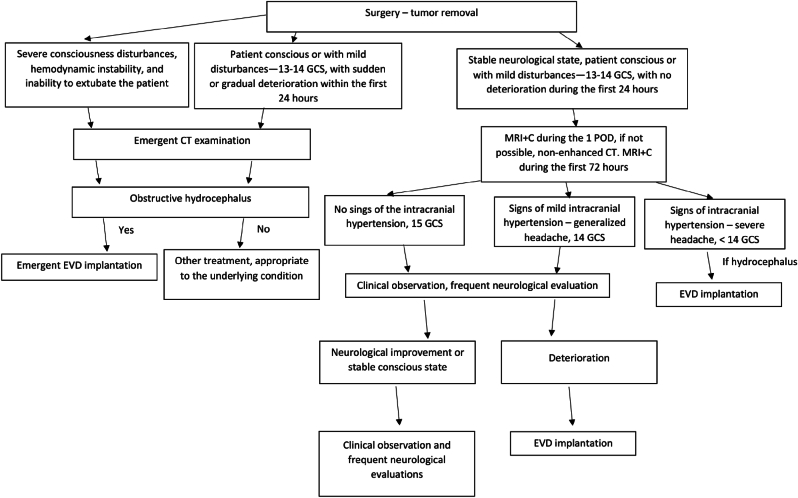
Step-by-step algorithm for diagnosing and managing early post-operative hydrocephalus following retrosigmoid vestibular schwannoma resection. GCS – Glasgow Coma Scale. EVD – external ventricular drainage.

Fig. 4 distills our step-by-step approach in the presentation of the postoperative hydrocephalus: CT or MRI examination in the 1st POD, routine contrast-enhanced MRI within 72 h if not performed in the 1st POD, trial of steroids (dexamethasone 4 mg every 12 h, escalated to every 8 h if needed) plus mannitol (15 %, 1 g per kg bolus followed by ≤ 200 g daily), neuromonitoring for 6–12 h, and EVD if GCS is less than 14 or there is no neurological improvement. This protocol is resource-sparing—only 5 % required EVD—and safe: no drain infections or tract hemorrhages occurred. Importantly, drains were removed on POD 5 after a 24-h clamp trial, illustrating that most cases reflect transient obstruction rather than irreversible CSF absorption failure.

The algorithm also impacts hospital metrics: EVD placement prolonged LOS by roughly one week (median 20 days vs 13 days). Early identification and decisive management thus have tangible cost and bed capacity implications that warrant inclusion in patient counseling and institutional planning.

Prospective, multi-institutional registries are needed to refine risk stratification, particularly the predictive value of intraoperative factors such as venous sacrifice, durotomy size, and posterior fossa closure technique. Advanced MRI techniques (e.g., phase-contrast CSF flow, diffusion tensor imaging) and serum biomarkers of blood–brain barrier disruption may help discriminate patients who are likely to fail medical therapy. Finally, controlled trials comparing short-course high-dose steroids with hypertonic saline could optimise first-line medical management.

## 5 Limitations

This retrospective, single-centre study may not be generalisable. The low incidence of hydrocephalus (eight symptomatic cases) limits statistical power and multivariable analysis. Imaging timing varied, and intracranial pressure was not monitored, so that transient events may have been missed. Finally, the median 22-month follow-up captures early outcomes but cannot exclude very late CSF-diversion procedures.

## 6 Conclusions

Early post-operative hydrocephalus after retrosigmoid VS surgery is invariably obstructive and presents in two clinical forms. Mild cases respond to short-term CSF diversion or medical therapy, whereas acute cases have a variable prognosis depending on the underlying lesion. Hematoma prevention and vigilant early imaging are pivotal to reducing morbidity.

## Author contributions

K.K. conceived the study, analysed the data and drafted the manuscript. M.T. and Ł.Sz. contributed to data analysis and interpretation. M.B., J.F. and M.H. critically revised the manuscript. All authors approved the final version.

## Funding

None.

## Declaration of competing interest

The authors declare that they have no known competing financial interests or personal relationships that could have appeared to influence the work reported in this paper.
